# The outcomes of post-traumatic arthritis vs osteoarthritis following primary total knee arthroplasty: A protocol of matched cohort study

**DOI:** 10.1097/MD.0000000000020077

**Published:** 2020-05-08

**Authors:** Jin-Quan Li, Hao-Xu, Ze-Gan Sun, Qing-Song Huang, Xiao-Dong Yao

**Affiliations:** Department of Orthopedics, 900TH Hospital of Joint Logistics Support Force, FuZhou, China.

**Keywords:** complication, osteoarthritis, post-traumatic arthritis, revision, study protocol, total knee arthroplasty

## Abstract

**Background:**

Total knee arthroplasty (TKA) for treatment of end-stage post-traumatic arthritis (PTA) has specific technical difficulties and complications. The aim of this study was to examine the outcome of TKA after PTA and to compare it with a cohort osteoarthritis (OA).

**Methods:**

A retrospective review of patients undergoing primary TKA at a single university hospital from 2013 to 2016 was performed. A minimum follow-up of 4 years was required. Patients in the study group were matched 1:2 with patients in the cohort group based on the following criteria: age at time of TKA (±3 years), body mass index (±3 points), sex, and American Society of Anesthesiologists score (±1 point). Outcome measures included surgical time, intraoperative complications, Oxford Knee Score, range of motion, postoperative complications, and revision.

**Results:**

This clinical trial is expected to determine whether PTA is associated with increased risks of complications and revision or reduced functional outcomes following TKA.

**Trial registration:**

This study protocol was registered in Research Registry (researchregistry5455).

## 1 Introduction

Total knee arthroplasty (TKA) is a popular procedure with excellent outcomes for most patients. The rise in incidence of osteoarthritis has resulted in an increase in the total annual volume of TKA in the United States.^[1]^ An estimated 1.4 million primary TKA procedures will occur in 2019.^[2]^ By 2030, the demand for primary TKA is projected to grow to 3.48 million procedures.^[2]^ The quest to improve patient outcomes is never-ending. Therefore, the imminent demand for TKA reinforces the importance of analyzing indications, patient populations, comorbidities, and outcomes.

Osteoarthritis (OA) is the primary indication for TKA, accounting for a vast majority of the cases. Outcomes following TKA in OA patients have been studied extensively. However, there are few studies which examine the outcomes of TKA in the setting of post-traumatic arthritis (PTA). PTA of the knee is a common complication of intra- or extra-articular fracture of the knee, with incidence estimated to be from 21% to 44%.^[3]^ Direct intra-articular injury, inadequate reduction or fixation of the fracture, residual malalignment, young patients, and previous joint degeneration are considered to be major risk factors for PTA.^[4]^ TKA on the PTA presents a number of technical considerations. Residual malalignment, scar tissue, stiffness, and reduced range of motion, a compromised soft tissue envelope secondary to the initial trauma, retained hardware, and secondary osseous deficiency represent a few of the technical challenges encountered while performing TKA on a posttraumatic knee.^[5,6]^ However, these technical challenges in TKA for PTA secondary to periarticular fracture are often similar to those seen in revision surgery including bone stock loss, although the reported complication rate in revision TKA is relatively lower.

Conflicting results have been reported concerning TKA in PTA. Several publications have reported high complication rates and less favorable functional outcomes in PTA compared with OA,^[5,7–8]^ whereas the other studies have found excellent results in young patients who have had TKA for secondary PTA.^[9–11]^ We thus designed a matched cohort study to compare PTA with OA patients in TKA. We hypothesized that there would be no difference in the patient reported outcome measures, complications or the revision rates between patients with PTA and OA undergoing TKA.

## 2 Material and method

### 2.1 Patient selection

A retrospective review of patients undergoing primary TKA at a single university hospital from 2013 to 2016 was performed. A minimum follow-up of 4 years was required. This study was approved by the institutional review board in our hospital (CZ2020102) and was registered in the research registry (researchregistry5455). The inclusion criteria were set as follows:

1. PTA or OA of the knee requiring primary TKA during hospitalization;
2. patients over the age of 40 and could cooperate with us for treatment and postoperative observation;
3. full demographic and follow-up data.

Exclusion criteria included the patient was pregnant or trying to become pregnant, cognitive impairment, a history of alcoholism, or if the patient was to undergo bilateral, simultaneous TKA. Patients in the study group were matched 1:2 with patients in the cohort group based on the following criteria: age at time of TKA (±3 years), body mass index (±3 points), sex, and American Society of Anesthesiologists score (±1 point).

### 2.2 Surgical technique

The surgical procedure was protocolized for both groups. All operations were performed under general anesthesia in an operating room with laminar flow. A standard anterior midlineskin incision and medial parapatellar arthrotomy was used in all patients. The Multigen (Lima, San Daniele, Italy) knee system was used in both groups, which was standard modular condylar-type prosthesis including posterior cruciate ligament-retained (CR, hybrid fixation), posterior-stabilized (PS, cemented fixation of both components) designs, and constrained PS design. Depending on the ligament balance at the time of surgery, a CR or PS design was used. Stem tibial component was always used to improve component fixation. In case of collateral ligament insufficiency, a constrained PS design with tibial and femoral stems was used. In both groups, the patella was systematically resurfaced with a three-pegged, all-polyethylene dome-shaped cemented design. Four specimens of synovial fluid and intraarticular tissue were taken intraoperatively at the time of TKA for microbiological analysis in all knees.

### 2.3 Postoperative care

The same postoperative protocol was used for both groups. Antibiotic prophylaxis was with third generation cephalosporin for 24 postoperative hours and thromboprophylaxis with low molecular weight heparin for 30 postoperative days. Continuous passive knee motion started on the first postoperative day, and beginning the third day active motion. In the patients who required a tibial tubercle osteotomy, passive motion was not used and active motion was limited to flexion 50° for 45 days. Early weight bearing as tolerated was encouraged in all patients.

### 2.4 Outcomes evaluations

The patient demographics, American Society of Anesthesiologists Score (ASA) grade, and body mass index (BMI) were recorded retrospectively from their electronic patient notes. Outcome measures included surgical time, intraoperative complications, Oxford Knee Score (OKS), range of motion (ROM), postoperative complications, and revision. Intraoperative complications and surgery time were obtained from review of the electronic medical records. The OKS, ROM, postoperative complications, and revision were obtained both before and after surgery at a minimum of 3 years postoperatively. Subjects were asked to complete an OKS questionnaire and rate their satisfaction with their TKA. The OKS consists of 12 questions assessed on a Likert scale with values from 0 to 4, a summative score is then calculated where 48 is the best possible score (least symptomatic) and 0 is the worst possible score (most symptomatic). For patients who were not seen recently, the scores were obtained via telephone. All data were independently verified by a detailed review of hospital operative reports, anesthesia records, and clinical records. Data were abstracted by 1 of 2 research personnel blinded to patient group and study aim. The results will be showed in Tables 1 to 3.

**Table 1:**
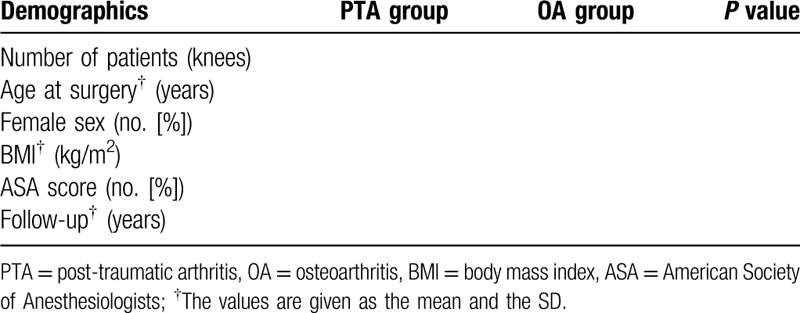
Patient baseline demographics.

**Table 2:**
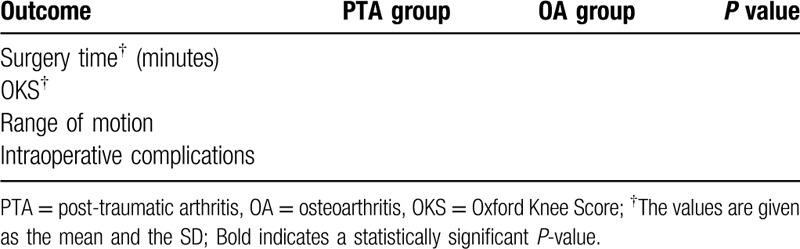
Intraoperative and functional outcomes.

**Table 3:**
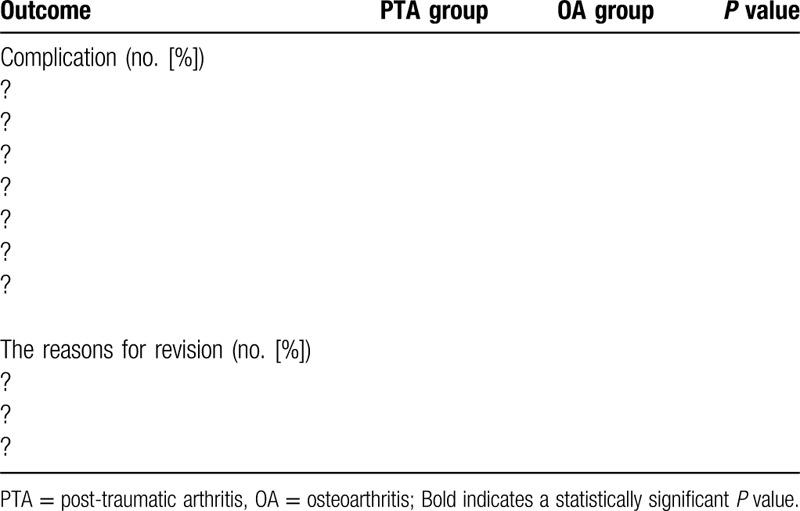
The complications and revision in the 2 groups.

### 2.5 Statistical Analysis

All statistical analyses are performed using SPSS v. 24 (IBM Corp., Armonk, NY, USA). The characteristics of patients, results of OKS, ROM, and surgery were reported as mean (SD). Qualitative variables were compared using chi-squared test and quantitative variables were compared with Student *t* test. OKS and ROM were compared between groups at last follow-up. The Mann–Whitney test for matched-pair comparisons was used to analyze results of OKS and ROM. Any *P* value <.05 was considered to be statistically significant. In survivorship analysis, Mantel-Cox log rank was used to assess significance during the construction of all Kaplan–Meyer curves. Confidence intervals (CIs) at the 95% level were determined.

## 3 Discussion

PTA of the knee is a common complication of intra- or extra-articular fracture of the knee, with incidence estimated to be from 21% to 44%.^[12]^ There are conflicting views concerning the best management of fractures around the knee, but it is generally accepted that articular irregularity, misalignment of the lower limb, and joint instability are the leading causes of PTA.^[13,14]^ In end-stage PTA, when nonoperative treatment has failed, TKA is a valid option. In PTA, surgeons have to deal with technical difficulties including previous scars, possible history of infection, misalignment related to malunion, stiffness, and sometimes ligament imbalance.^[15]^ Thus, performing TKA in PTA is more challenging than in primary osteoarthritis. Several studies on this subject have been published without conclusive results. We thus designed a matched cohort study to compare PTA with OA in TKA.

The limitations of our study included those inherent in any retrospective cohort study, including the possibility of selection or observational bias. This study also did not address long-term follow-up (10 years) as our study relied on electronic medical records kept since 2013. The authors recognize that longer term follow-up is critical in determining the influence of PTA on TKA specifically on infection, implant loosening, revision, and long-term function outcomes. Additionally, although we performed a matched study based on age, gender, American Society of Anesthesiologists score, and body mass index, it is likely that there were other pre-operative features that we could have controlled for that may have led to alternative results. This clinical trial is expected to determine whether PTA is associated with increased risks of complications and revision or reduced functional outcomes following TKA.

## Author contributions

Jin-Quan Li and Hao-Xu conceived, designed, and planed the study. Ze-Gan Sun and Qing-Song Huang are recruiting the study participants and performing the interventions. Xiao-Dong Yao supervised the study. Jin-Quan Li and Xiao-Dong Yao will interpret and analyze the data. Jin-Quan Li drafted the manuscript. Jin-Quan Li and Hao-Xu critically revised the manuscript for important intellectual content. All authors have full access to the manuscript and take responsibility for the study design. All authors have approved the manuscript and agree with submission.
